# Serial Assessment of Cardiac Function during and following Mitoxantrone Infusion in 30 Consecutive Patients with Multiple Sclerosis

**DOI:** 10.1155/2010/351045

**Published:** 2010-12-01

**Authors:** Damian Franzen, Angelika Haus, Martin Hellmich

**Affiliations:** ^1^Berrenratherstr. 296, 50937 Köln, Germany; ^2^Durenerstr. 332, 50935 Köln, Germany; ^3^Institute of Medical Statistics and Epidemiology, University of Cologne, 50924 Köln, Germany

## Abstract

Immunosuppressive therapy is an established therapeutic option in patients suffering from multiple sclerosis (MS). In an open nonrandomized study we serially assessed cardiac function in 30 consecutive patients with MS before, during, and after mitoxantrone therapy. Mitoxantrone (12 mg/m^2^) was administered intravenously at 3-month intervals. Before each infusion, cardiac function was assessed by history taking, resting electrocardiogram, and echocardiography. Whereas no patient experienced clinical signs of heart failure, left ventricular pump function decreased continuously during mitoxantrone therapy and did not recover after cessation. The presented data suggest a dose-dependent and long-lasting toxic cardiac effect of low-dose mitoxantrone therapy in MS.

## 1. Introduction

In the year 2003 immunosuppressive therapy using mitoxantrone has been approved for treatment of patients with worsening relapsing-remitting or secondary progressive multiple sclerosis (MS) in Germany. Due to potential side effects on the heart and bone marrow the cumulative dose of mitoxantrone was initially limited to 100 mg/m^2^ and later on extended to 140 mg/m^2^ in selected cases.

However, systematic data on the course and incidence of cardiac toxicity following low-dose mitoxantrone are lacking. In the following, we report about serial cardiac evaluation in 30 consecutive patients before, during, and after mitoxantrone treatment in consecutive patients with MS. In the years 2006 and 2008, parts of the study have been published in German language in journals which are not indexed by PubMed [1, 2].

## 2. Methods

From January 2003 to July 2004, thirty consecutive patients with secondary progressive or worsening relapsing multiple sclerosis were treated with mitoxantrone at 3-month intervals. Treatment was initiated and terminated at the discretion of the attending neurologist. It was agreed among the participating physicians that termination of treatment due to cardiac reasons was limited to clinical heart failure only. Mitoxantrone infusion as well as cardiac evaluation before, during, and after treatment was performed by a cardiologist. All patients were treated ambulatory and received a pneumococcal vaccination before treatment was initiated.

In the following we focus on the cardiac evaluation of mitoxantrone infusion. Prior to each treatment, all patients underwent a careful history taking, laboratory testing, a resting electrocardiogram (ECG), and a transthoracic cardiac ultrasound. On all occasions, mitoxantrone at a dose of 12 mg/m^2^ diluted in 250 ml saline was infused over a period of 30 minutes. 

The electrocardiogram was obtained by a digital 12-channel system (Custo Cardio 130, Ottobrunn) in recumbent position. Heart rate was averaged over a period of 30 seconds. 

Cardiac imaging was performed using a digital ultrasound system (Vivid 3, General Electrics, Solingen). Cardiac chamber measurements (mm), calculation of fractional shortening (FS%), and respective ejection fraction (EF%) were done on M-mode images only; values were determined as an average of three consecutive measurements. Global assessment of cardiac function and valvular disease was performed on two-dimensional views.

## 3. Statistics

The relation between EF, FS, LVEDD, and heart rate with the cumulative mitoxantrone dose was analyzed using linear regression analysis with random coefficients (software SAS 9.1, proc mixed). The course of each patient was linearly modeled and the population average (trend) estimated respecting age and gender. *P* values less than 5% were considered statistically significant.

## 4. Results

30 consecutive patients (23 female, 7 male) with a mean age of 47 ± 10 years were treated with mitoxantrone. No patient had received mitoxantrone, a mediastinal radiation, or any cancer treatment before the present mitoxantrone therapy. Three patients suffered from noninsulin-dependent diabetes (10%), 2 patients from essential hypertension (7%), and 4 patients from depression. No patient suffered from renal insufficiency. Prior to mitoxantrone treatment 8 patients had been treated with interferon (27%), 3 with azathioprine (10%), 1 with glatiramer acetate (3%), 2 with interferon and azathioprine (7%), 1 with glatiramer acetate and interferon (3%), and 1 with immunoglobulins (3%).

In 4/30 patients mitoxantrone therapy had been started during hospitalization elsewhere (9 courses) and continued mitoxantrone treatment as all other 26 patients ambulatory. All reported data refer to the data obtained in all 30 patients during ambulatory treatment only. Mitoxantrone was infused 222 times with a mean cumulative dosage of 83  ± 37 mg/m^2^ (minimal dose 24 mg/m^2^, maximal 156 mg/m^2^). Treatment frequency ranged from 2 to 13 infusions per patient and extended over a mean period of 23 ± 13 months (minimal 3, maximal 53 months). 

During therapy with mitoxantrone 3 patients (10%) experienced an acute episode of relapsing symptoms, one after the first course, one after the 4th, and one after the 5th course of mitoxantrone. In two of these 3 patients, an additional high-dose course of intravenous steroids was administered. At the terminal mitoxantrone infusion 5/30 (17%) reported about amelioration of MS symptoms as compared to symptoms at the start of therapy, 19/30 (63%) about no change, and 6/30 (20%) about worsening symptoms.

Out of 30 treated patients a total of 19 patients (63%) had one or more cardiac exams following termination of mitoxantrone infusion. Nine out of 19 patients (47%) had only one followup. The followup extended over a mean period of 30 ± 14 months (minimal 6, maximal 56 months).

### 4.1. Cardiac Evaluation

At the start of mitoxantrone therapy, history taking and a careful exam revealed no cardiac disease in any patient. During and after mitoxantrone treatment no patient developed cardiac failure or was hospitalized for any reason.

Resting ECG was normal before, during, and after mitoxantrone treatment in all patients. Mean heart rate averaged 77 ± 11 rpm before treatment and did not change significantly during and after mitoxantrone treatment (Table 1).

**Figure 1:**
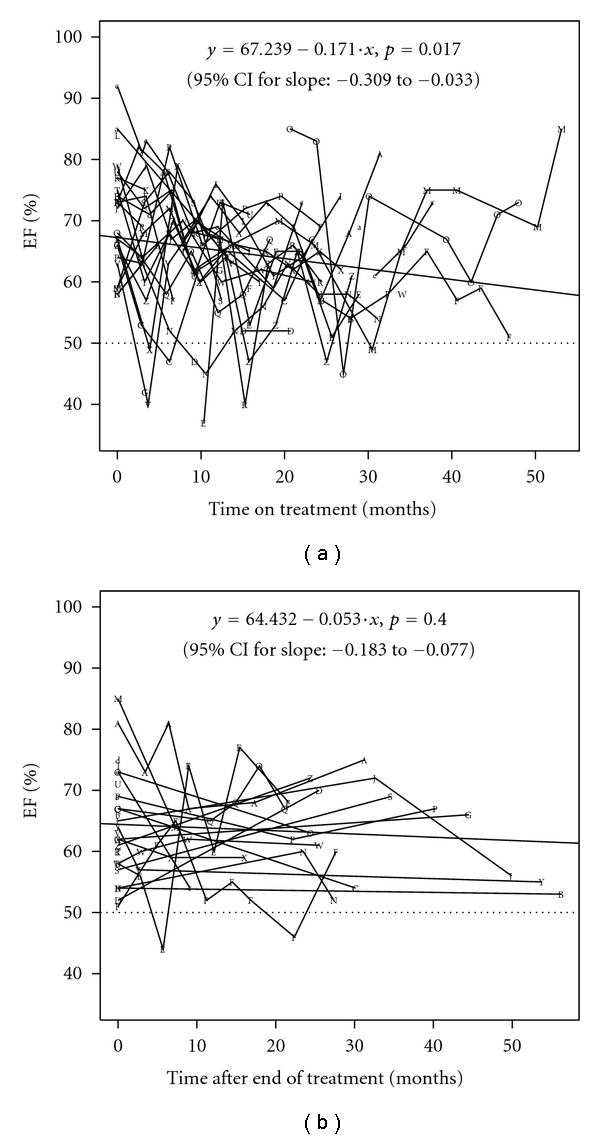
(a) Time course of left ventricular ejection fraction (EF%) during mitoxantrone treatment of 19 patients. In several patients, time on treatment was prolonged due to temporary interruptions of treatment. The time course of individual patients is denoted by the same letter throughout Figures 1(a) and 1(b). (b) Time course of left ventricular ejection fraction (EF%) after the end of mitoxantrone treatment in the same 19 patients is shown in Figure 1(a).

Whereas heart rate and left end diastolic dimension did not change significantly during and after therapy, fractional shortening (FS) as well as ejection fraction (EF) decreased continuously and statistically significantly during mitoxantrone treatment in the study population of 30 patients (Table 1). During followup of 19 patients, cardiac pump function as determined by FS and EF did not recover (Figures 1(a) and 1(b) for 19 patients during and after treatment). In contrast, 2D echocardiography did not reveal any visible deterioration of global left ventricular function. No patient developed valvular problems or signs of endocarditis.

## 5. Discussion

Mitoxantrone therapy is an established option for the treatment of multiple sclerosis [3]. Apart from therapy-related acute leukemia, cardiac pump failure is considered as the most serious side effect [3, 4]. According to published reports mitoxantrone cardiotoxicity occurs infrequently; however, this problem has not been assessed systematically. In some studies cardiac function and respective ejection fraction have been evaluated by various different methods [5]. In other smaller studies, cardiac function has been addressed only before and at the end of treatment leaving the results subject to alpha and beta errors [6]. In the present study, cardiac function has been explored systematically and serially allowing for longitudinal intraindividual observations of cardiac function. In 30 patients treated with mitoxantrone at a mean dosage of 83 ± 37 mg/m^2^, left ventricular pump function decreased continuously without clinical signs of heart failure. Following termination of treatment, cardiac function did not recover, suggesting a long-lasting toxic effect of mitoxantrone on the heart. Our findings are supported by publications of Avasarala et al. [7] and Pattoneri et al. [8] but contrast with studies of De Castro et al. [9] and Zingler et al. [10] who found no decline in left ventricular function during serial assessment using echocardiography. 

Based on numerous reports of early and late onset cardiotoxicity in individuals with low-dose mitoxantrone and our observations of dose-dependent effects on the heart, serial determination of cardiac function in each patient seems indicated. Although cardiac pump function and respective ejection fraction can be determined by various methods, transthoracic echocardiography is cost-effective and available everywhere; radionuclide studies or magnetic resonance imaging should be reserved to selected patients with questionable results. Whether ejection fraction is calculated linearily using M-mode echocardiograms or volumetrically using planimetry of monoplane or biplane projections in patients with suspected nonischemic cardiomyopathy is of minor importance as long as the reevaluation monitoring employs the same method throughout the course of chemotherapy and its followup. Apart from the evaluation of systolic cardiac pump function by echocardiography, repetitive careful history taking and the serial determination of brain natriuretic petide may guide the followup [11]. Parameters of impaired relaxation and respective abnormalities in diastolic filling of the heart may precede reductions in systolic pump function and respective ejection fraction, in patients receiving chemotherapy [12].

On the basis of the current paper, we suggest to inform the patient about the potential insidious nature of cardiac toxicity and to monitor cardiac function in every patient prior and, systematically, during mitoxantrone therapy. It seems that cardiac function does not deteriorate further when mitoxantrone therapy has stopped. In future studies, it should be tested by longitudinal serial analysis whether pretreatment with dexrazoxane will limit the slow decline of left ventricular function as has been reported by Bernitsas et al. [13].

## Figures and Tables

**Table 1:** Mean values for heart rate, left ventricular enddiastolic dimension (LVEDD), ejection fraction (EF), and fractional shortening (FS). Standard deviation and number of patients in parenthesis. *Pvalue ≤ .05.

	Treatment start	At cessation of treatment	At the beginning of follow-up	At the end
Heart rate (rpm)	77 (12, 28)	76 (16, 30)	76 (14, 19)	73 (13, 19)
LVEDD (mm)	46 (5, 25)	44 (5, 27)	45 (5, 19)	46 (5, 19)
EF (%)	71 (9, 24)	65 (9, 27)*	63 (6, 19)	62 (7, 19)
FS (%)	41 (8, 24)	36 (6, 26)*	33 (5, 19)	33 (5, 19)
